# Indicate separate contributions of long-lived and short-lived greenhouse gases in emission targets

**DOI:** 10.1038/s41612-021-00226-2

**Published:** 2022-01-28

**Authors:** Myles R. Allen, Glen P. Peters, Keith P. Shine, Christian Azar, Paul Balcombe, Olivier Boucher, Michelle Cain, Philippe Ciais, William Collins, Piers M. Forster, Dave J. Frame, Pierre Friedlingstein, Claire Fyson, Thomas Gasser, Bill Hare, Stuart Jenkins, Steven P. Hamburg, Daniel J. A. Johansson, John Lynch, Adrian Macey, Johannes Morfeldt, Alexander Nauels, Ilissa Ocko, Michael Oppenheimer, Stephen W. Pacala, Raymond Pierrehumbert, Joeri Rogelj, Michiel Schaeffer, Carl F. Schleussner, Drew Shindell, Ragnhild B. Skeie, Stephen M. Smith, Katsumasa Tanaka

**Affiliations:** 1 School of Geography and the Environment and Department of Physics, University of Oxford, Oxford, UK; 2 CICERO Centre for International Climate Research, Oslo, Norway; 3 Department of Meteorology, University of Reading, Reading, UK; 4 Chalmers University of Technology, Göteborg, Sweden; 5 Queen Mary University of London, London, UK; 6 Institute Pierre Simon Laplace, Gif-sur-Yvette, France; 7 Cranfield University, Bedford, UK; 8 Laboratoire des Sciences du Climat et de l’Environnement, Gif-sur-Yvette, France; 9 University of Reading, Reading, UK; 10 University of Leeds, Leeds, UK; 11 Victoria University of Wellington, Wellington, New Zealand; 12 University of Exeter, Exeter, UK; 13 Climate Analytics, Berlin, Germany; 14 International Institute for Applied Systems Analysis (IIASA), Vienna, Austria; 15 University of Oxford, Oxford, UK; 16 Environmental Defence Fund, New York, NY, USA; 17 Princeton University, Princeton, NJ, USA; 18 Imperial College, London, UK; 19 Duke University, Durham, NC, USA

As researchers who have published over recent years on the issue of comparing the climate effects of different greenhouse gases, we would like to highlight a simple innovation that would enhance the transparency of stocktakes of progress towards achieving any multi-decade-timescale global temperature goal. In addition to specifying targets for total CO_2_-equivalent emissions of all greenhouse gases, governments and corporations could also indicate the separate contribution to these totals from greenhouse gases with lifetimes around 100 years or longer, notably CO_2_ and nitrous oxide, and the contribution from Short-Lived Climate Forcers (SLCFs), notably methane and some hydrofluorocarbons. This separate indication would support an objective assessment of the implications of aggregated emission targets for global temperature, in alignment with the UNFCCC Parties’ Decision (4/ CMA.1)^1^ to provide “information necessary for clarity, transparency and understanding” in nationally determined contributions (NDCs) and long-term low-emission development strategies (LT-LEDSs).

While differences remain between us regarding how best to set fair yet ambitious targets for individual emitters^2–5^, including how any additional information might be used, and the interpretation of the Paris Agreement, it is important to emphasise the high level of agreement on the underlying science of how different greenhouse gases affect global temperature. The 2018 IPCC Special Report on 1.5 °C (SR1.5)^6^ stated “Reaching and sustaining net-zero global anthropogenic CO_2_ emissions and declining net non-CO_2_ radiative forcing (Planetary energy imbalance resulting directly from human-induced changes.) would halt anthropogenic global warming on multi-decadal timescales (*high confidence*). The maximum temperature reached is then determined by cumulative net global anthropogenic CO_2_ emissions up to the time of net zero CO_2_ emissions (*high confidence*) and the level of non-CO_2_ radiative forcing in the decades prior to the time that maximum temperatures are reached (*medium confidence*)”. The IPCC 6th Assessment Report (AR6)^7^ confirmed “limiting human-induced global warming to a specific level requires limiting cumulative CO_2_ emissions, reaching at least net zero CO_2_ emissions, along with strong reductions in other greenhouse gas emissions”.

Parties to the Paris Agreement agreed in Katowice in 2018 (Decision 18/CMA.1)^1^ to report past emissions of individual gases separately and use 100-year Global Warming Potentials (GWP_100_) when aggregating them to CO_2_-equivalent (we refer to these here as CO_2_-e_100_ emissions). The separate specification of individual gases minimises ambiguity in determining the climate impact of past emissions. NDCs and other future targets are, however, almost always expressed in terms of aggregate CO_2_-e_100_ emissions only, for which the implications for global temperature are ambiguous^8,9^. Separate specification of the contribution from CO_2_ helps, but ambiguity in global temperature outcomes remains if targets for non-CO_2_ gases comprise a mixture of long-lived climate forcers (LLCFs), such as nitrous oxide, with atmospheric lifetimes around 100 years or longer, and SLCFs, such as methane, most of which have lifetimes shorter than 20 years^10^.

Specifying the contributions of all gases individually in future targets as well as the reporting of past emissions would resolve the ambiguity in global temperature outcomes, and would also help quantify non-climate benefits of emission reductions, especially for methane^11^. Governments and particularly corporations may, however, wish to retain some level of aggregation across gases to allow flexibility in how they achieve their targets. Fortunately, a much less restrictive approach delivers almost all the transparency benefits from a climate perspective. The climate system responds similarly over a broad range of timescales to equal emissions expressed in tonnes of CO_2_-e_100_ of all LLCFs, including CO_2_^12^. Likewise, the net radiative forcing due to SLCFs on multi-decadal timescales is similar to the aggregated rate of SLCF emissions expressed in tonnes of CO_2_-e_100_ per year multiplied by the 100-year Absolute Global Warming Potential (AGWP_100_) of CO_2_^13^. With this additional information, it is straightforward to express the SR1.5 statement quoted above in terms of CO_2_-e_100_ emissions: human-induced warming over any multi-decade time-interval is approximately the sum of (i) aggregate CO_2_-e_100_ emissions of LLCFs, including CO_2_, multiplied by a constant parameter, the Transient Climate Response to cumulative CO_2_ Emissions, or TCRE^14^ (the TCRE can alternatively be thought of as the Absolute Global Temperature-Change Potential for a sustained emission of CO_2_ divided by the time-horizon, AGTP_s_/H^13^); (ii) any change in decadal-average radiative forcing due to SLCFs multiplied by another constant parameter, the Transient Climate Response to Forcing, or TCRF, another name for the “fast” component(s) of the climate response^15^; and (iii) a gradual adjustment to average SLCF forcing^16^, all evaluated over the same time-interval.

Hence a separate indication of the contributions of LLCFs and SLCFs in emission targets, or equivalently the LLCF contribution to total CO_2_-e_100_ emissions, is required to allow for the global temperature outcome to be calculated relatively unambiguously. It is important to note, however, that the evaluation of emission targets at the national or corporate level cannot be undertaken from a physical science perspective alone, but also depends on economic, social, equity and political considerations^2–5,17^, including responsibility for past warming, capacity for and costs of abatement, and non-climate impacts. Separate specification would also facilitate the use of alternate or flexible emission metrics, which may be useful for achieving a cost-effective emission trajectory over time^18^ or addressing specific policy goals such as limiting near-term rates of warming^19^. Indicative contributions from LLCF and SLCF abatement would not preclude trade-offs between them, but would clarify the need to monitor the temperature impacts of any such trade-offs over a range of timescales^20^.

It has long been accepted^21^ that stringent mitigation of both LLCFs and SLCFs is needed to meet any ambitious temperature goal, but making progress on two fronts necessitates monitoring progress on two fronts. Some countries (but very few companies) already specify the contribution of LLCFs and/or SLCFs to total CO_2_-e_100_ emissions in NDCs, LT-LEDSs and science-based targets (https://sciencebasedtargets.org/) communicated under the Greenhouse Gas Protocol. Quantifying the aggregated implications of these targets for future global temperature simply requires a much wider uptake of this practice, representing a simple and achievable innovation that would enhance the transparency of any stocktake of progress towards any global temperature outcome. Separate indication of LLCF and/or SLCF contributions could be communicated by countries as additional information consistent with Decision 4/CMA.1. This does not have to affect any existing or planned NDCs or long-term net zero strategies^22^ communicated using aggregate CO_2_-e_100_.

## Why Separate Specification is so Useful

To quantify the SR1.5 and AR6 statements quoted above, human-induced global temperature change over a multi-decade time-interval Δ*t*, relative to the level of human-induced warming at the beginning of that interval (e.g. the present day or pre-industrial), can be decomposed using the framework articulated above as follows:

(1) $$\text{Δ}T=\kappa_{E}\bar{E_{C}}\text{Δ}t+\kappa_{F}\left(\text{Δ}F_{N}+\rho \bar{F_{N}}\text{Δ}t\right),$$

where $\bar{E_{C}}$ and $\bar{F_{N}}$ are globally aggregated average CO_2_ emissionrates and non-CO_2_ radiative forcing, respectively (so $\bar{E_{C}}\text{Δ}t$ is cumulative CO_2_ emissions), and Δ*F_N_* is the change in decadal- average non-CO_2_ forcing, all evaluated over that interval (the geophysical “Zero Emissions Commitment” is expected to be relatively small over a multi-decade time-interval^23^, but this may not be the case on longer timescales). The coefficients *K_E_* (the TCRE) and *K_F_* (the TCRF, or “fast” component of the climate response to any forcing change, denoted *C*_1_ in ref. ^12^, or sum of fast components^24^: see supplementary material), are both scenario-independent in the absence of strongly non-linear carbon cycle feedbacks or climate response. The only scenario-dependent coefficient is *ρ*, the fractional Rate of Adjustment to Constant Forcing (RACF), or the relatively small fractional rate at which forcing needs to decline to maintain stable temperatures. It depends on how fast and how recently *F_N_* has increased (this term represents the delayed adjustment to past forcing increases, so is larger for more recent and rapid increases). If *F_N_* varies only on multi-decadal timescales, *ρ* = *C*_2_/(*K_F_S*_2_), where *C*_2_ is the “slow” (multi-century) component of the climate sensitivity, and *S_2_* the deep ocean thermal adjustment timescale. For representative^12^ coefficient values, *ρ* ≤ 0.3% per year, making this third term usually small.

Aggregate CO_2_-e_100_ emissions cannot be used to calculate *F_N_* if these comprise a mixture of LLCFs and SLCFs. Aggregate CO_2_-e_100_ emissions of LLCFs, *E_L_*, can, however, be combined unambiguously and have the same impact on global temperature on decade to century timescales as the corresponding quantity of CO_2_. Likewise, aggregate CO_2_-e_100_ emissions of SLCFs, *E_S_*, multiplied by the AGWP_100_ of CO_2_, *A*_100_, give SLCF radiative forcing, *F_S_* (*A*_100_ normally includes a first-order estimate of the impact of carbon cycle feedbacks^25^ so, for consistency, this should also be included in the GWP_100_ values used to compute *E_S_*).

For emissions reported as CO_2_-e_100_ the above expression can therefore be re-written (now grouping all LLCFs with CO_2_):

(2) $$\text{Δ}T=\kappa_{E}\bar{E_{L}}\text{Δ}t+\kappa_{F}\left(\text{Δ}F_{S}+\rho \bar{F_{S}}\text{Δ}t\right),$$

or equivalently, using *F_S_* = *A*_100_ *E_S_* on multi-decadal timescales,

(3) $$\text{Δ}T=\kappa_{E}\bar{E_{L}}\text{Δ}t+\kappa_{F}A_{100}\left(\text{Δ}E_{S}+\rho \bar{E_{S}}\text{Δ}t\right).$$

Hence Δ*T* can be estimated directly using well-known (albeit uncertain) climate system properties if, and only if, total CO_2_-e_100_ emissions of long-lived climate forcers, *E_L_*, are specified in emission targets together with total CO_2_-e_100_ emissions, *E_L_* + *E_S_*; or, equivalently, *E_L_* and *E_S_* are specified separately. Δ*T* cannot be calculated from the sum of *E_L_* + *E_S_* alone.

**Fig. 1:**
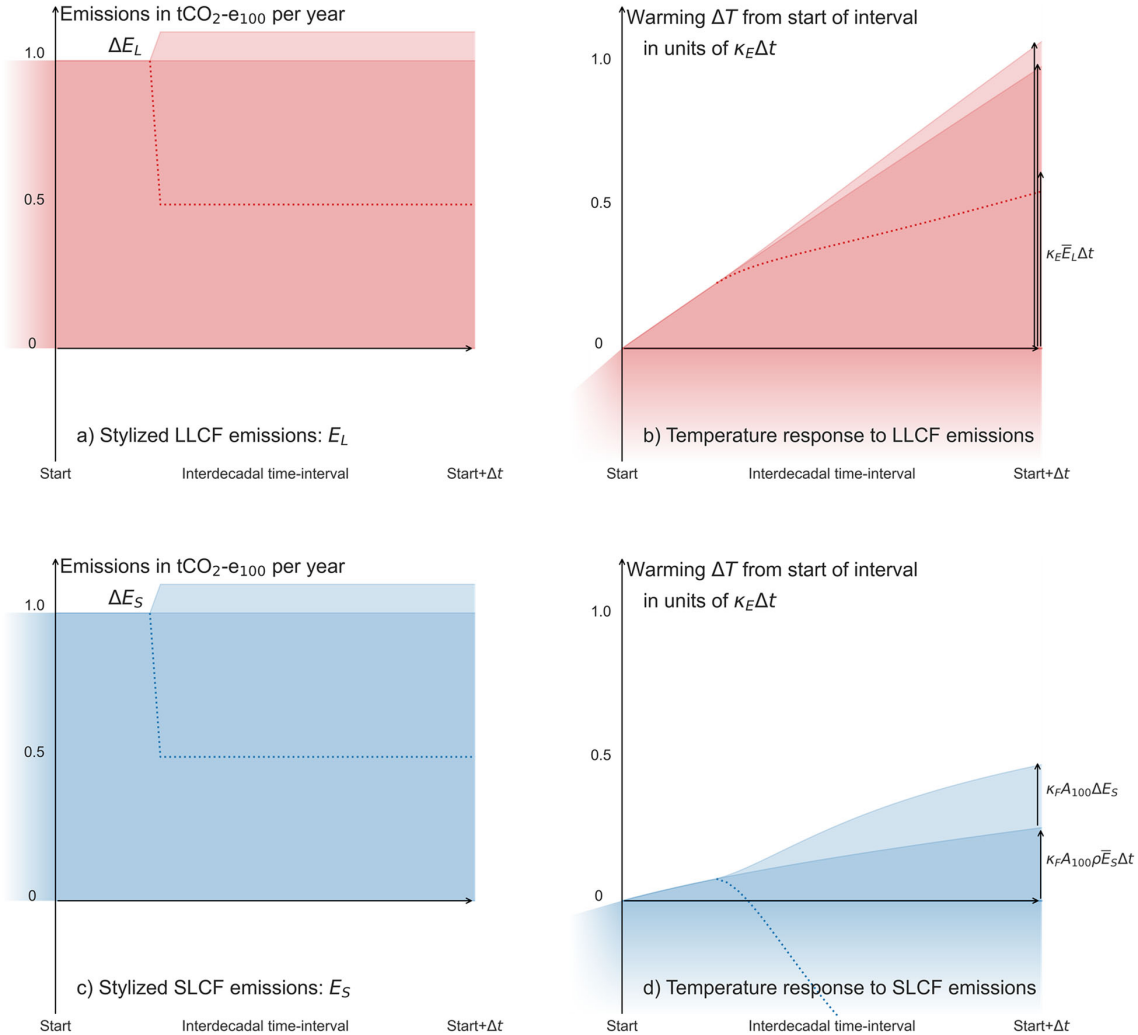
Stylised LLCF and SLCF emissions and resulting global temperature change Δ*T* over a multi-decade period. Darker bands in panels **a** and **c** show, respectively, constant LLCF and SLCF emissions of 1 tCO_2_-e_100_ per year starting some decades before the interval shown. Pale bands show a 10% increase one-quarter of the way through the interval shown, while dotted lines show a 50% decrease. Resulting temperature changes relative to the start of this interval shown in panels **b** and **d**, calculated using a simple climate model: vertical axes in **b** and **d** are scaled identically to illustrate smaller rate of warming due to constant SLCF emissions and much larger warming impact of any change in SLCF emissions relative to the warming due to identical CO_2_-e_100_ LLCF emissions. Vertical arrows in the right show predicted contributions to Δ*T* from the individual terms in Eq. (3): three arrows in panel **b** show cumulative LLCF emissions over this interval multiplied by the TCRE for the three scenarios shown; the lower and upper arrows in panel **d** show, respectively, the predicted warming due to ongoing constant SLCF emissions and additional warming due to the 10% increase. The figure illustrates that Eq. (3) allows reliable, if approximate, prediction of multi-decade warming Δ*T* if, and only if, LLCF and SLCF emissions are specified separately.

This is illustrated by Fig. 1, which shows the impact of LLCF and SLCF emissions, expressed as CO_2_-e_100_, on global temperature change over a multi-decade period, relative to the level of warming at the beginning of that period, calculated with a simple climate model^12^. Stylised cases of constant (darker shades) and step-change (+10%, lighter shades, and −50%, dotted lines) emissions are shown in panels a and c. Warming due to LLCF emissions (the term $K_{E}\bar{E_{L}}\text{Δ}t$ in Eq. (3)) increases linearly with cumulative emissions in all three cases (panel b). Warming due to an ongoing constant emission of an SLCF that started decades before the beginning of this period (the $K_{F}A_{100}\rho \bar{E_{S}}\text{Δ}t$ term) also increases linearly (panel d, darker blue) but at a slower rate per tCO_2_-e_100_ emitted (by a factor of about 4, because *K_E_* ≈ 4 × *K_F_A*_100_ *ρ*): global temperatures have already partially equilibrated with this constant emission (by how much depends on how long ago these SLCF emissions began, which is why *ρ* is the only scenario-dependent coefficient in these expressions). Finally, warming due to an increase in SLCF emissions (the *K_F_A*_100_Δ*E_S_* term, panel d, lighter blue) is 4–5 times greater than would be expected from the same increase in tCO_2_-e_100_ emissions of an LLCF (panel b, lighter red) over the 20 years following the increase (*K_F_A*_100_ ≈ 4.5 × *K_E_* × 20 years). Hence the AR6 statement “expressing methane emissions as CO_2_ equivalent emissions using GWP_100_ overstates the effect of constant methane emissions on global surface temperature by a factor of 3–4 … while understating the effect of any new methane emission source by a factor of 4–5 over the 20 years following the introduction of the new source”^26^ applies to the impact of global emissions of any SLCF. Any decrease in SLCF emissions also has a much greater impact on temperatures over a multi-decade period per tCO_2_-e_100_ avoided than a corresponding decrease in LLCF emissions (red and blue dotted lines) (Fig. 1).

Temperature changes in the figure are calculated using a particular model, LLCF, SLCF and scenario. The figure would, however, appear similar if another model, combination of gases or scenario of prior emissions were used, provided emissions do not change rapidly immediately before the beginning or end of the period shown, because the relationship between emissions and warming expressed in Eq. (3) is generic. Individual terms in Eq. (3), assuming constant coefficients, are shown by the arrows on the right of panels b and d. These match the warming calculated by the explicit simple climate model within modelling uncertainties. The figure shows temperature change relative to the start of the period rather than absolute warming because the latter is not determined by Eq. (3) but depends on the prior LLCF and SLCF emissions history (the specific scenario used to generate this figure is shown in full in the Supplementary Information).

Temperature change Δ*T* over a multi-decade period depends, to first order, only on cumulative emissions of LLCFs $\bar{E_{L}}\text{Δ}t$ , cumulative emissions of SLCFs $\bar{E_{S}}\text{Δ}t$ , and net change in total SLCF emission rates Δ*E_S_*, over that period alone. As the SR1.5 and AR6 emphasised, future warming depends on future emissions. Making use of this information, however, requires both *E_L_* and *E_S_* to be specified: only specifying the sum *E_L_* + *E_S_* introduces an ambiguity in temperature outcome.

Separate specification also facilitates assessing the implications of different metrics. For example, aggregate CO_2_-equivalent emissions using the 20-year Global Warming Potential (GWP_20_) can be approximated by *E_L_* + 3*E_S_* if both *E_L_* and *E_S_* are reported as CO_2_-e_100_, with a slightly higher multiplicative factor (up to 4) if *E_S_* is dominated by forcers with lifetimes of order one year (Table 8.A.1 of ref. ^12^ shows that GWP_20_ values are similar to GWP_100_ values for LLCFs and 3 or 4 times GWP_100_ values for gases with lifetimes of order a decade or a year, respectively). Finally, we re-emphasise that these expressions capture our physical understanding of how global emissions of LLCFs and SLCFs collectively determine global temperature change, and illustrate the utility of separate specification of *E_L_* and *E_S_*. How this understanding is used to inform the assessment of the adequacy of individual emission targets depends on other considerations listed above and cannot be argued from a physical science perspective alone. There will be several other advantages to the additional communication such as being able to estimate air quality co-benefits of mitigation.

## Supplementary Material

**Supplementary information** The online version contains supplementary material available at https://doi.org/10.1038/s41612-021-00226-2.

S1

## Data Availability

Data sharing is not applicable to this article as no datasets were generated or analysed during the current study. A self-contained Python notebook to reproduce the figure is provided on https://gitlab.ouce.ox.ac.uk/OMP_climate_pollutants/separate-contributions.
