# Supplementary material for: Indicate separate contributions of long-lived and short-lived greenhouse gases in emission targets
Source: NPJ Clim Atmos Sci. Author manuscript; Available in PMC 2022 Mar 15. (PMC7612487; doi:10.1038/s41612-021-00226-2)
Supplement: S1 [file EMS143808-supplement-S1.docx]

## Indicate separate contributions of long-lived and short-lived greenhouse gases in emission targets

Myles R Allen, Glen P Peters, Keith P Shine, *et al*

**Supplementary information**

**Derivation of global temperature change equations**

The temperature response to a forcing timeseries $F\left( t \right)$ is (8.SM.12 and 8.SM.13 of ref. ^[[1]](#endnote-1)^):

$$T\left( t \right)=\sum_{j=1}^{M} \frac{c_{j}}{s_{j}}\int_{t^{'}=0}^{t} F\left( t^{'} \right)\exp\left( -\frac{t-t^{'}}{s_{j}} \right)dt^{'}$$

where the $c_{j}$ are $M$ components of the climate response in °C per W/m^2^, $\sum_{i} c_{i}=\text{ECS}/{F_{2\times}}$, the Equilibrium Climate Sensitivity divided by the forcing due to doubling CO_2_, and the $s_{j}$ are response times. For a ramp forcing increase, $F\left( t \right)=gt$, where $g$ is a constant rate of increase. Using the integral $\int_{0}^{t} t^{'}exp\left( \left( t-t^{'} \right)/s \right)dt^{'}=s\left( t-s\left( 1-\exp\left( -t/s \right) \right) \right)$ we have:

$$T\left( t \right)=g\sum_{j=1}^{M} c_{j}\left[ t-s_{j}\left( 1-\exp\left( -\frac{t}{s_{j}} \right) \right) \right] .$$

If $M=2$, $s_{1}$ is of order years and $s_{2}$ of order centuries, as in ref. 1, then on timescales such that $s_{1}\ll t$, $\exp\left( {-t}/{s_{1}} \right)\approx0$, while if $t\ll s_{2}$ then $\exp\left( {-t}/{s_{2}} \right)\approx1-t/{s_{2}}+{t^{2}}/\left( {2s}_{2}^{2} \right)$. Hence:

$$T\left( t \right)\approx g\left[ c_{1}\left( t-s_{1} \right)+\frac{c_{2}t^{2}}{2s_{2}} \right] .$$

Thus the temperature change over a multi-decade time-interval in response to a linear forcing ramp starting from zero consists of one component proportional to the forcing increase over that time-interval (calculating $\Delta F$ between the decade prior to the beginning and the decade prior to the end of the time-interval accounts for the small delay $s_{1}$, so $\Delta F=g\left( \Delta t-s_{1} \right)$) and a second component proportional to ${\Delta t}^{2}$, or the average forcing multiplied by the length of the time-interval, $\bar{F}\Delta t={g{\Delta t}^{2}}/2$:

$$\Delta T=\kappa_{F}\left( \Delta F+\rho\bar{F}\Delta t \right)$$

where $\kappa_{F}=c_{1}\approx0.5$ °C per W/m^2^ and $\rho={c_{2}}/\left( c_{1}s_{2} \right)\approx\left( 300 \text{years} \right)^{-1}$, which is also the fractional rate of adjustment to constant forcing (RACF) following a multi-decade increase. Since this expression is linear, it also applies to any $F\left( t \right)$ that can be expressed as a superposition of such interdecadal ramps. It also applies for $M>2$ provided there is only one centennial response time, all other response-times are sub-decadal and $c_{1}$ is replaced by the sum of the sub-decadal components.

**Model calculations used in the figure**

The figure in the main text shows temperature changes $\Delta T$ over a multi-decade interval of duration $\Delta t$ due to cumulative emissions of LLCFs $\bar{E_{L}}\Delta t$, cumulative emissions of SLCFs $\bar{E_{S}}\Delta t$, and net change in total SLCF emission rates $\Delta E_{S}$, over this interval. To first order, the appearance of the figure is the same, and the correspondence with the individual terms in the equations illustrated by the vertical arrows is the same, whatever model, specific LLCF and SLCF, and prior emissions history are used to calculate the figure and for any multi-decade $\Delta t$, $\bar{E_{L}}$, $\bar{E_{S}}$ and $\Delta E_{S}$ provided emissions do not change substantially in the decades immediately prior to the beginning and end of the interval shown. A choice has to be made, however, to generate the figure: we use the standard IPCC impulse response model^1^ with an equilibrium climate sensitivity of 3°C and thermal time-constants of 4.1 and 249 years^[[2]](#endnote-2)^ and all gas properties following ref. 1, giving a TCRE 0.47 °C/TtCO_2­_ based on years 30-100, a TCRF 0.48 °C/(W/m^2^), an AGWP_100_ for CO_2_ of 92 W-years/m^2^/TtCO_2­_ and $\rho=\left( 366 \text{years} \right)^{-1}$. The model is forced with constant 1 GtCO_2_/year CO_2_ emissions and 1 GtCO_2_-e_100_/year methane emissions for 200 years, the figure in the main text shows years 50-90 as a representative multi-decade period. Full emission history and temperature response are illustrated below.


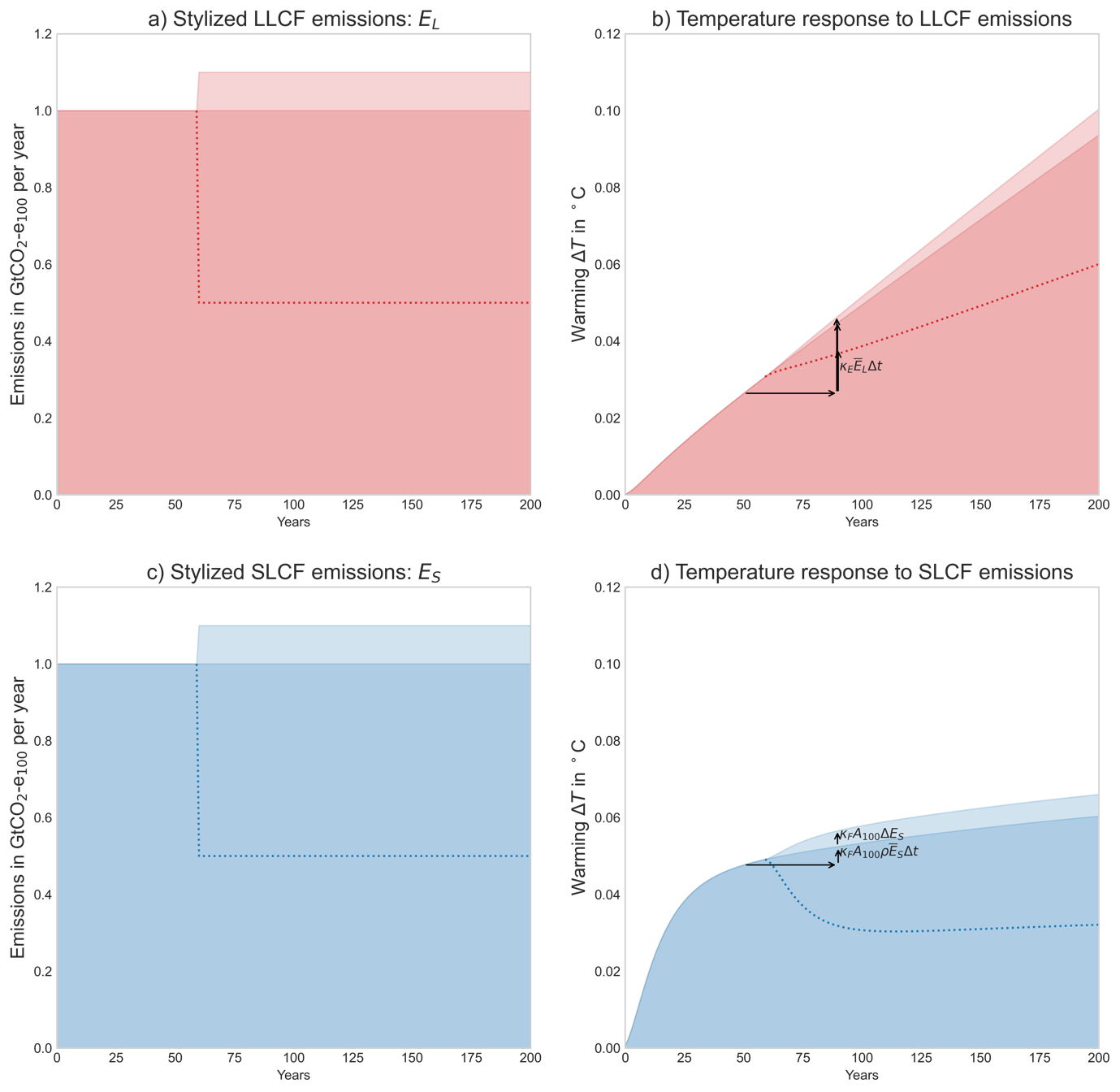


**Supplementary Figure 1:** Full emissions history and temperature response used to generate the schematic figure in the main text. Left panels show constant emissions of (a) CO_2_ and (c) methane for 200 years, starting in year 0, increasing by 10% (lighter shade) and decreasing by 50% (dotted) in year 60. Right panels show temperature response using the IPCC linear impulse-response model, with arrows illustrating temperature changes over the interval years 50 to 90 shown in the figure in the main text. Python code to reproduce this figure and that in the main text accompanies this supplementary information.

1. Myhre, G., D. Shindell et al. Anthropogenic and Natural Radiative Forcing, Ch. 8 of Stocker, T., D. Qin et al (eds), Climate Change 2013: Scientific Basis, Contr. of WG1 to the IPCC Fifth Assess. Rep., Cambridge, (2013). [↑](#endnote-ref-1)
2. Geoffroy, O. et al. Transient Climate Response in a Two-Layer Energy-Balance Model. Part I: Analytical Solution and Parameter Calibration Using CMIP5 AOGCM Experiments. *J. Clim.* **26**, 1841–1857 (2012). [↑](#endnote-ref-2)
